# The Evolution and Diversity of SALMFamide Neuropeptides

**DOI:** 10.1371/journal.pone.0059076

**Published:** 2013-03-11

**Authors:** Maurice R. Elphick, Sufyan Achhala, Natalia Martynyuk

**Affiliations:** Queen Mary University of London, School of Biological & Chemical Sciences, London, United Kingdom; Goethe University Frankfurt, Germany

## Abstract

The SALMFamides are a family of neuropeptides that act as muscle relaxants in echinoderms. Two types of SALMFamides have been identified: L-type (e.g. the starfish neuropeptides S1 and S2) with the C-terminal motif LxFamide (x is variable) and F-type with the C-terminal motif FxFamide. In the sea urchin *Strongylocentrotus purpuratus* (class Echinoidea) there are two SALMFamide genes, one encoding L-type SALMFamides and a second encoding F-type SALMFamides, but hitherto it was not known if this applies to other echinoderms. Here we report the identification of SALMFamide genes in the sea cucumber *Apostichopus japonicus* (class Holothuroidea) and the starfish *Patiria miniata* (class Asteroidea). In both species there are two SALMFamide genes: one gene encoding L-type SALMFamides (e.g. S1 in *P. miniata*) and a second gene encoding F-type SALMFamides plus one or more L-type SALMFamides (e.g. S2-like peptide in *P. miniata*). Thus, the ancestry of the two SALMFamide gene types traces back to the common ancestor of echinoids, holothurians and asteroids, although it is not clear if the occurrence of L-type peptides in F-type SALMFamide precursors is an ancestral or derived character. The gene sequences also reveal a remarkable diversity of SALMFamide neuropeptides. Originally just two peptides (S1 and S2) were isolated from starfish but now we find that in *P. miniata*, for example, there are sixteen putative SALMFamide neuropeptides. Thus, the SALMFamides would be a good model system for experimental analysis of the physiological significance of neuropeptide “cocktails” derived from the same precursor protein.

## Introduction

Secreted neuropeptides mediate intercellular neuronal signalling and are involved in the regulation of diverse physiological processes and behaviours [1]. To gain knowledge and understanding of how neuropeptide signalling systems have evolved it is necessary to analyse neuropeptide systems in a wide range of animal phyla. However, research on neuropeptides in invertebrates has largely focused on protostomes, which include model organisms such as *Drosophila melanogaster* (Phylum Arthropoda), *Caenorhabditis elegans* (Phylum Nematoda) and *Aplysia californica* (Phylum Mollusca) [2], [3]. Much less is known about neuropeptide systems in deuterostomian invertebrates, which include invertebrate chordates (cephalochordates and urochordates), hemichordates and echinoderms (starfish, sea urchins, sea cucumbers). Nevertheless, research on these animals has recently begun to provide important insights on the evolution of neuropeptide signalling systems [4]. Furthermore, the pentaradial body plan of adult echinoderms provides a unique context for investigation of the physiological roles of neuropeptide signalling systems [4], [5].

The first echinoderm neuropeptides to be identified were the SALMFamide neuropeptides S1 and S2, which were both isolated from the starfish species *Asterias rubens* and *Asterias forbesi* on account of their cross-reactivity with antibodies to the molluscan FMRFamide-like neuropeptide pQDPFLRFamide [6], [7]. S1 is a C-terminally amidated octapeptide with the amino acid sequence Gly-Phe-Asn-Ser-Ala-Leu-Met-Phe-NH_2_ (GFNSALMFamide) and S2 is a C-terminally amidated dodecapeptide with the amino acid sequence Ser-Gly-Pro-Tyr-Ser-Phe-Asn-Ser-Gly-Leu-Thr-Phe-NH_2_ (SGPYSFNSGLTFamide). Comparison of the sequences of S1 and S2 revealed that both peptides have the C-terminal motif FNSxLxFamide (where x is variable), suggesting that S1 and S2 may have evolved as a consequence of gene duplication or intragenic DNA duplication [6].

Subsequently, antibodies to pQDPFLRFamide were also used to monitor purification of SALMFamide neuropeptides from another echinoderm species, the sea cucumber *Holothuria glaberrima* [8]. Two structurally related neuropeptides were identified: Gly-Phe-Ser-Lys-Leu-Tyr-Phe-NH_2_ (GFSKLYFamide) and Ser-Gly-Tyr-Ser-Val-Leu-Tyr-Phe-NH_2_ (SGYSVLYFamide). Furthermore, comparison of the sequences of these sea cucumber neuropeptides with the starfish SALMFamides S1 and S2 revealed a shared C-terminal motif: SxLxFamide (where x is variable). Thus, it was postulated that this motif may be a defining characteristic of a family of SALMFamide neuropeptides in the phylum Echinodermata [8].

The discovery of SALMFamides enabled the first investigations of the physiological roles of identified neuropeptides in echinoderms. *In vitro* studies revealed that S1 and S2 both cause relaxation of cardiac stomach, tube foot and apical muscle preparations from *A. rubens*, but with S2 being more potent/effective than S1 [9], [10], [11]. Furthermore, the relaxing effects of S1 or S2 *in vivo* cause eversion of the cardiac stomach, indicating that S1 and/or S2 may mediate physiological control of stomach eversion during feeding in starfish [10]. Consistent with this notion, antibodies to S1 and S2 reveal immunoreactive nerve fibres in close association with the muscle layer of the cardiac stomach [12]. Likewise, S1- and/or S2-immunoreactive fibres are also present in the tube feet and apical muscle, indicative of a general role for these peptides as regulators of muscle relaxation in starfish [13]. Furthermore, the two SALMFamides identified in *H. glaberrima* also cause relaxation of intestinal and longitudinal body wall muscle preparations in this species [14]. Thus, it appears that SALMFamides may act as muscle relaxants throughout the phylum Echinodermata [15].

The muscle-relaxing effect of SALMFamide neuropeptides was also observed when longitudinal body wall muscle and intestine preparations were used as bioassays to screen for myoactive peptides in the sea cucumber *Apostichopus japonicus*. A total of twenty myoactive peptides were identified but only two of these were found to cause muscle relaxation: GYSPFMFamide and FKSPFMFamide [16]. It was clear that these two peptides are structurally related to the SALMFamide neuropeptides previously identified *H. glaberrima* - GFSKLYFamide and SGYSVLYFamide. However, the presence in the *A. japonicus* peptides of a phenylalanine (F) residue in the position occupied by a leucine (L) residue in the *H. glaberrima* peptides broadened the structural motif of SALMFamide neuropeptides to Sx(L/F)xFamide. It remained unclear though whether the identification of SALMFamides in different sea cucumber species that either have a SxLxFamide motif (L-type SALMFamides) or have a SxFxFamide motif (F-type SALMFamides) reflects species-specific variation in SALMFamide structure or whether L-type and F-type SALMFamides coexist in echinoderm species. This issue was resolved with identification of two SALMFamide genes in the sea urchin *Strongylocentrotus purpuratus* – one gene that encodes a precursor protein comprising seven putative F-type SALMFamide neuropeptides (SpurS1– SpurS7) and another gene that encodes a precursor protein comprising two putative L-type SALMFamide neuropeptides (SpurS8– SpurS9) [17], [18]. This discovery demonstrated that L-type and F-type SALMFamides do both occur in an echinoderm species but are derived from different precursor proteins. Recently, however, a gene encoding SALMFamide neuropeptides in the sea cucumber *A. japonicus* has been identified [19], revealing a precursor protein that comprises eight putative neuropeptides, including the two F-type SALMFamides originally identified in this species on account of their muscle relaxing effects (GYSPFMFamide and FKSPFMFamide), as well as two L-type SALMFamides that share sequence similarity with the two L-type SALMFamides originally isolated from *H. glaberrima*. Thus, L-type and F-type SALMFamides coexisting in an echinoderm species can also be derived from the same precursor.

A number of questions remain to be answered with regard to the phylogenetic distribution and evolution of SALMFamide neuropeptides in the Phylum Echinodermata. Does the existence of a gene in *A. japonicus* encoding a precursor comprising both L-type and F-type SALMFamides reflect a characteristic of the common ancestor of the Holothuroidea (sea cucumbers) and the Echinoidea (sea urchins), two sister classes within the phylum Echinodermata? If it does, then the existence in *S. purpuratus* of two genes encoding precursors comprising either L-type or F-type SALMFamides could be explained by duplication of a gene encoding the putative ancestral-type precursor followed by specialisation as either L-type or F-type precursors. However, it is noteworthy that the SALMFamide precursor previously identified in *A. japonicus* [19] is quite similar to the F-type SALMFamide precursor in *S. purpuratus* [17] in at least two respects. Firstly, with respect to the number of putative neuropeptides derived from the precursors; the *S. purpuratus* precursor contains seven putative neuropeptides and the *A. japonicus* precursor contains eight putative neuropeptides. Secondly, whilst the *A. japonicus* precursor does contain putative L-type SALMFamides, it is predominantly an F-type SALMFamide precursor with five out of eight putative neuropeptides having an F-type motif. Because of this similarity between the previously reported *A. japonicus* SALMFamide precursor and the *S. purpuratus* F-type SALMFamide precursor, we postulate that there may be a second SALMFamide precursor in *A. japonicus* that is related to the L-type SALMFamide precursor in *S. purpuratus.*

Another issue that remains to be addressed is the phylogenetic distribution of F-type SALMFamides. Thus far, F-type SALMFamides have only been identified in sea cucumbers and sea urchins. Do F-type SALMFamides also exist in other echinoderm classes – the Ophiuroidea (brittle stars), the Asteroidea (starfish) and the Crinoidea (sea lilies, feather stars)? Lastly, is the diversity of SALMFamides that have been identified by analysis of gene sequences in a sea urchin species (nine SALMFamides in *S. purpuratus*) [18] and a sea cucumber species (eight SALMFamides in *A. japonicus*) [19] indicative of the number of SALMFamides that exist in other echinoderms? Evidence that this may be the case, at least in starfish, was provided by the purification and sequencing of four SALMFamides from nerve cord extracts of *Marthasterias glacialis*. Thus, in addition to S1 and an S2-like peptide (SGPYSMTSGLTFamide; MagS2), two other SALMFamides were identified: MagS3 (AYHSALPFamide) and MagS4 (AYQTGLPFamide) [20]. Definitive determination of the number of SALMFamides that exist in a starfish species, however, will require sequencing of the gene(s) encoding these peptides.

To address the questions outlined above, we have investigated the occurrence of an L-type SALMFamide gene in *A. japonicus* and have searched for SALMFamide genes in the starfish species *Patiria miniata*. The data obtained provide important new insights on the phylogenetic distribution, evolution and structural diversity of SALMFamide neuropeptides in the phylum Echinodermata.

## Methods

### Apostichopus Japonicus

To search for a second SALMFamide precursor in *A. japonicus,* the *S. purpuratus* L-type SALMFamide precursor was submitted as a query in a tBLASTn search of 29,666 *A. japonicus* contigs, which were downloaded from the supporting information section (Dataset S1) of an online paper by Du et al. [21] available from the PloSONE website (http://www.plosone.org/article/info%3Adoi%2F10.1371%2Fjournal.pone.0033311). BLAST searches were performed using SequenceServer software (http://www.sequenceserver.com/), which is freely available to academic users [22].

### Patiria Miniata

To search for SALMFamide genes in the starfish *Patiria miniata*, genome sequence data generated by the Baylor College of Medicine Human Genome Sequencing Center were downloaded from NCBI (http://0-www.ncbi.nlm.nih.gov.ilsprod.lib.neu.edu/nuccore/399761416). Sequence data were downloaded as 179,756 contigs (AKZP01000001 - AKZP01179756) and as 21,465 scaffolds (JH768608 - JH790072) and then BLAST searches of the sequence data were performed using SequenceServer software.

### Comparative Analysis of the Sequences of SALMFamide Precursor Proteins

The online multiple sequence alignment tool Clustal Omega (http://www.ebi.ac.uk/Tools/msa/clustalo/; with default parameters) was used to enable comparative analysis of the sequences of echinoderm SALMFamide precursor proteins.

## Results and Discussion

### Identification of a Second SALMFamide Gene in *Apostichopus japonicus*

**Figure 1:**
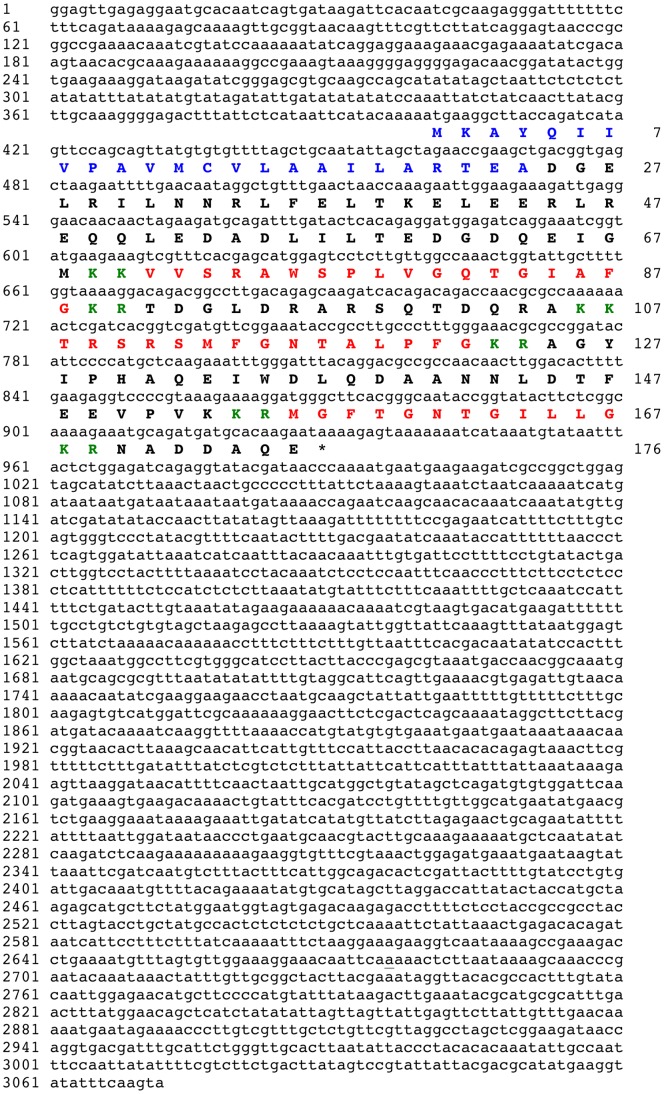
*Apostichopus japonicus* L-type SALMFamide precursor. The cDNA sequence (lowercase, 3072 bases) of isotig 08429, which encodes an L-type SALMFamide precursor protein (bold uppercase, 176 amino acid residues) is shown. The predicted signal peptide is shown in blue and the three putative SALMFamide neuropeptides are shown in red flanked by putative dibasic cleavage sites (KR or KK) shown in green. The asterisk shows the position of the stop codon.

When the protein sequence of the *S. purpuratus* L-type SALMFamide precursor [18] was submitted as a query in a tBLASTn search of *A. japonicus* isotig sequence data, the top two hits were isotig 08429 (isogroup 02114; 3072 bases) and isotig 08430 (isogroup 02114; 2838 bases). Comparative analysis of the sequences of isotigs 08429 and 08430 revealed that they share 99% nucleotide sequence identity and encode the same protein (see below), with the only major difference between them being that 08429 has a 3′ extension with respect to 08430. Therefore, the sequence of the longer isoform (08429) is shown in Figure 1.

Analysis of the sequences of isotigs 08429 and 08430 revealed that they encode a 176-residue protein comprising an N-terminal signal peptide and three putative L-type or L-type-like SALMFamide neuropeptides bounded by putative dibasic (KR, KK) endopeptidase cleavage sites (Figure 1). All three putative neuropeptides have a C-terminal glycine residue, which is likely to be a substrate for amidation [23]. The first putative neuropeptide is a 17-residue peptide, the second neuropeptide is a 14-residue peptide and the third neuropeptide is an 11-residue peptide. The 14-residue peptide (TRSRSMFGNTALPFamide) has the canonical L-type SALMFamide C-terminal LxFamide motif. In the 17-residue peptide (VVSRAWSPLVGQTGIAFamide) the leucine residue that characterizes L-type SALMFamides is replaced by the structurally related amino acid isoleucine and in this respect it is similar to the *S. purpuratus* L-type-like SALMFamide SpurS8, which has the C-terminal sequence IHFamide [18]. The C-terminal sequence of the 11-residue peptide (MGFTGNTGILLamide) is ILLamide, which is unusual because a leucine (L) residue replaces the C-terminal phenylalanine (F) residue that is characteristic of both L-type and F-type SALMFamide neuropeptides. Interestingly, a peptide (FKSSFYLamide) with the same modification is a predicted product of the SALMFamide precursor previously identified in *A. japonicus*, which is a precursor of both F-type and L-type SALMFamides [19].

### Identification of a Gene in *Patiria miniata* that Encodes S1 and Other L-type SALMFamides

**Figure 2:**
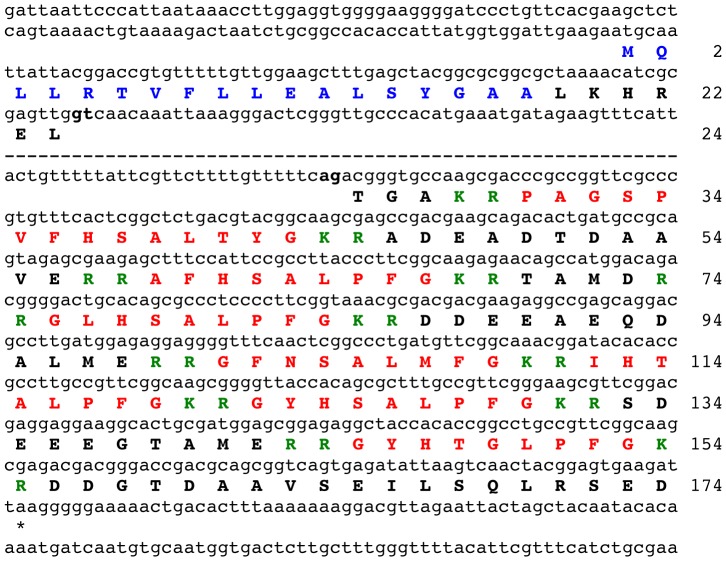
*Patiria miniata* L-type SALMFamide precursor. The sequence of a gene (lowercase) in *P. miniata* that encodes an L-type SALMFamide precursor protein (bold uppercase, 174 amino acid residues) is shown. The majority of a large intron that separates the two exons is not shown (dashed line) but the 5′ and 3′ regions are shown, including the respective splice sites gt and ag, which are highlighted in bold. The predicted signal peptide of the precursor protein is shown in blue and the seven putative SALMFamide neuropeptides are shown in red flanked by putative dibasic cleavage sites (KR or RR) shown in green. The asterisk shows the position of the stop codon.

To search for a gene encoding the starfish neuropeptide S1 and/or structurally related neuropeptides, a *P. miniata* contig dataset was queried (using tBLASTn) with KRGFNSALMFGKR, an S1-containing amino acid sequence bounded by dibasic cleavage sites. A single hit was identified (contig 39135; 29729 bases) and analysis of the sequence of contig 39135 revealed that it contains an open reading frame encoding a 150-residue polypeptide sequence comprising S1 and six other putative L-type SALMFamide neuropeptides. Because the 150-residue sequence lacked an N-terminal signal peptide it was inferred that the gene must have an additional exon or exons that encode the N-terminal signal peptide. To facilitate identification of this putative exon, the 150-residue sequence was submitted as a query in a tBLASTn search of the *P. miniata* genome scaffold dataset, which revealed that the coding sequence for the 150-residue polypeptide is in scaffold 1914 (55,595 bases). The sequence of scaffold 1914 was then analysed using gene prediction tools (GenScan: http://genes.mit.edu/GENSCAN.html; Augustus: http://bioinf.uni-greifswald.de/augustus/submission) but the predicted proteins incorporating the 150-residue sequence containing SALMFamide neuropeptides did not have an N-terminal signal peptide sequence. Therefore, the region of scaffold 1914 located 5′ to the exon encoding the 150-residue sequence containing SALMFamide neuropeptides was analysed systematically to identify an open reading frame that encodes a polypeptide with an N-terminal signal peptide. Using this strategy an exon encoding a signal peptide-containing polypeptide was successfully identified upstream from the SALMFamide-encoding exon, separated by an 18,144 base intron. Thus, the predicted precursor of S1 and other L-type SALMFamides in *P. miniata* is a 174-residue protein with an 18-residue N-terminal signal peptide (Figure 2).

### Identification of a Gene in *Patiria miniata* that Encodes F-type SALMFamides and an S2-like Peptide

**Figure 3:**
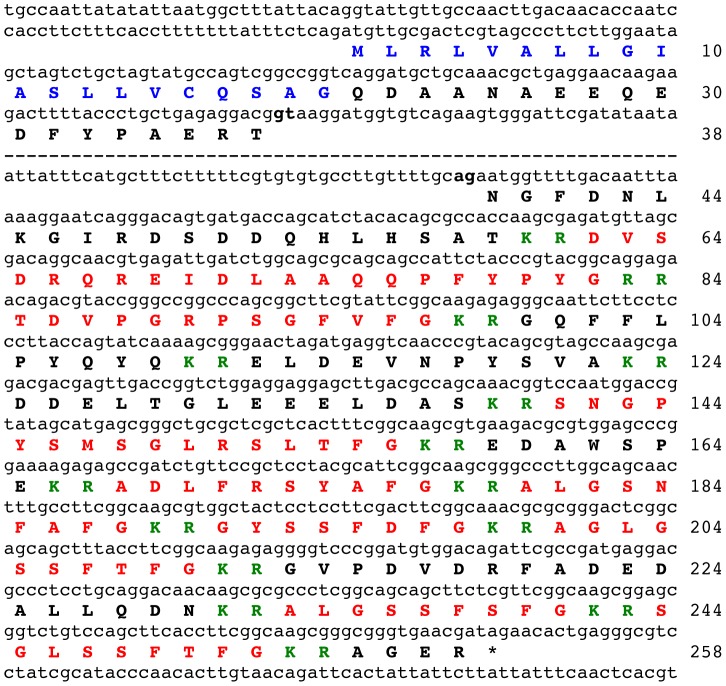
*Patiria miniata* F-type SALMFamide precursor. The sequence of a gene (lowercase) in *P. miniata* that encodes an F-type SALMFamide precursor protein (bold uppercase, 258 amino acid residues) is shown. The majority of a large intron that separates the two exons is not shown (dashed line) but the 5′ and 3′ regions are shown, including the respective splice sites gt and ag, which are highlighted in bold. The predicted signal peptide of the precursor protein is shown in blue and the nine putative SALMFamide neuropeptides are shown in red flanked by putative dibasic cleavage sites (KR or RR) shown in green. The asterisk shows the position of the stop codon.

As the SALMFamide precursor comprising S1 and other L-type SALMFamides (Figure 2) does not contain S2 or an S2-like peptide, it was deduced that there might be a second SALMFamide precursor in *P. miniata*. To search for this putative gene, the amino acid sequence of the *P. miniata* S1 precursor was submitted as a query in a tBLASTn search of the *P. miniata* contig dataset. This revealed the existence of a second SALMFamide gene on contig 45038 (27003 bases). Analysis of the sequence of contig 45038 using the gene prediction tool GenScan revealed the presence of a gene comprising two exons (separated by a 3318 base intron), with the first exon encoding a 38-residue sequence with an N-terminal signal peptide and the second exon encoding a 220 residue sequence comprising a putative S2-like peptide (SNGPYSMSGLRSLTFamide) and eight putative F-type or F-type-like SALMFamide neuropeptides (Figure 3). Thus, in *P. miniata* an S2-like L-type SALMFamide neuropeptide is derived from a 258-residue precursor of F-type SALMFamides.

### The Evolution of SALMFamide Neuropeptide Precursors

**Figure 4:**
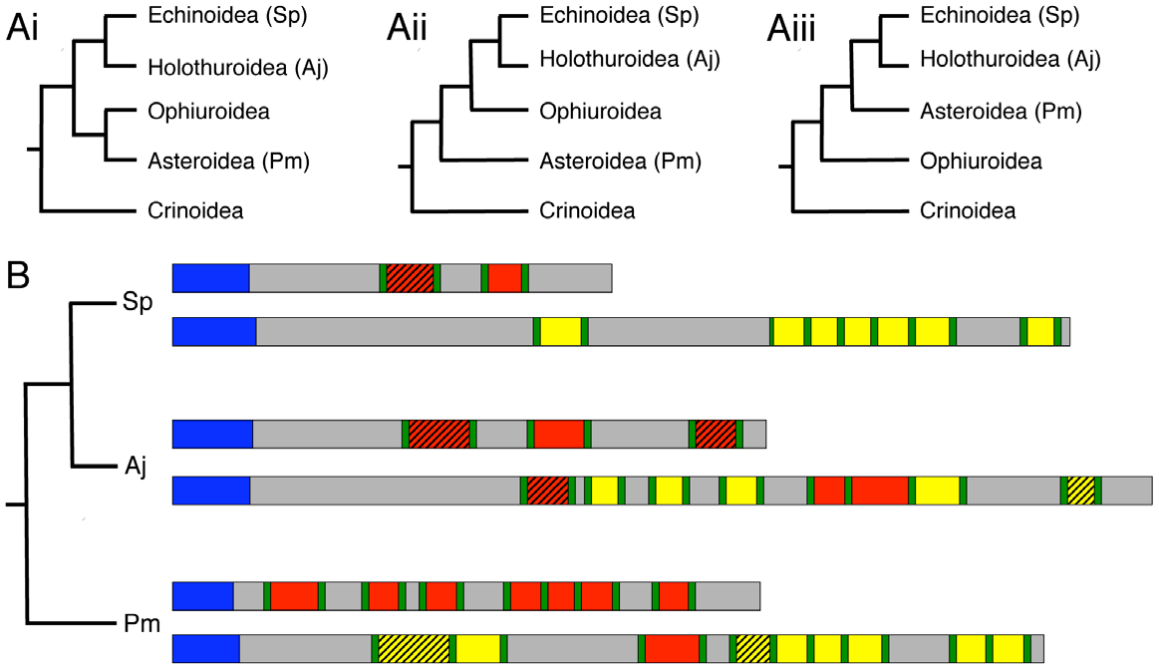
Phylogenetic analysis of SALMFamide precursors in echinoderms. **A.** Echinoderm phylogeny. The basal position of crinoids and the sister group status of echinoids and holothurians is widely accepted but there is conflicting evidence with respect to the phylogenetic position of asteroids and ophiuroids [24]; therefore the three possible echinoderm phylogenies are shown in Ai, Aii and Aiii. **B.** Phylogenetic diagram showing the occurrence and organisation of SALMFamide precursors in species representing three echinoderm classes: the Echinoidea (*Strongylocentrotus purpuratus*; Sp), the Holothuroidea (*Apostichopus japonicus*; Aj) and the Asteroidea (*Patiria miniata*; Pm). Signal peptides are shown in blue and dibasic or monobasic cleavage sites are shown in green. L-type SALMFamides with the canonical C-terminal LxFamide motif are shown in red and peptides that are “L-type-like” (e.g. MGFTGNTGILLamide in *A. japonicus*, where the L and F are replaced by I and L, respectively) are shown in red with hatched shading. Likewise, F-type SALMFamides with the canonical C-terminal FxFamide motif are shown in yellow and peptides that are “F-type-like” (e.g. ADLFRSYAFamide in *P. miniata*, where one of the F residues is replaced by Y) are shown in yellow with hatched shading. In each species (class) there are two types of SALMFamide precursor: Firstly, a precursor that is exclusively comprised of L-type peptides or L-type-like peptides. Secondly, a precursor that is either exclusively comprised of F-type peptides (Sp) or a precursor that is largely comprised of F-type peptides or F-type-like peptides together with one or more L-type or L-type-like peptides (Aj and Pm).

The identification of SALMFamide neuropeptide precursors in species from three different echinoderm classes has provided the first opportunity to investigate the evolution of SALMFamide neuropeptides, informed by current thinking on the phylogenetic relationships of the five extant echinoderm classes [24]. There is consensus that crinoids are basal with respect to the four other extant classes, with a divergence time estimated at ∼ 509 million years ago. There is also consensus that echinoids and holothurians are sister groups. However, there is conflicting evidence with respect to the position of the asteroids and ophiuroids and evidence for all three possible groupings (Figure 4A) has been obtained [24]. This difficulty in resolving relationships between the echinoid/holothurian, asteroid and ophiuroid clades is attributed to their divergence over a relatively short period of geological time (∼ 5 million years) 475–480 million years ago [24]. Nevertheless, regardless of which of the alternate phylogenies (Figure 4A) is correct, with the sequence data now available it is possible to draw several conclusions on the phylogenetic distribution and evolution of SALMFamide precursors.

In Figure 4B the three pairs of SALMFamide precursors that have been identified in species representing three echinoderm classes are shown in a phylogenetic schematic. What is immediately apparent from this diagram is that in each class (as represented by one species) there are two types of SALMFamide precursor: Firstly, a precursor that is exclusively comprised of L-type peptides or L-type-like peptides. Secondly, a precursor that is either exclusively comprised of F-type peptides (as in the echinoid species) or a precursor that is largely comprised of F-type peptides or F-type-like peptides together with one or more L-type or L-type-like peptides (as in the holothuroid and asteroid species). We can infer from this that it is likely that the common ancestor of echinoids, holothurians and asteroids would have had two SALMFamide genes: one encoding only L-type SALMFamides (an “L-type SALMFamide precursor”) and a second encoding exclusively or largely F-type SALMFamides (an “F-type SALMFamide precursor”). What remains uncertain based on the data available is whether the presence of L-type peptides in F-type SALMFamide precursors reflects the ancestral condition or whether it is a derived condition. The former requires invoking loss of L-type peptides in F-type SALMFamide precursors in only one lineage, the echinoids, whereas the latter requires invoking independent acquisition of L-type peptides in the F-type SALMFamide precursors of two lineages, the holothurians and the asteroids. It is not possible draw firm conclusions based on the data currently available but further insight on this issue will be obtained if SALMFamide genes are identified in an ophiuroid species. Moreover, as the most basal of the five extant classes [24], it will be especially interesting to identify a SALMFamide gene or genes in a crinoid species. Thus, does the occurrence of the two SALMFamide gene types date back to the common ancestor of all extant echinoderm classes? Alternatively, do crinoids have only a single L/F-type SALMFamide gene?

Clearly there is much still to be learnt about the evolutionary history of SALMFamide genes. However, even with data from just three echinoderm species, some general characteristics of SALMFamide genes/precursors are apparent. For example, it is evident from the two species where genome sequence data are available (*S. purpuratus* and *P. miniata*) that SALMFamide genes comprise two protein-coding exons: the first exon encoding the predicted N-terminal signal peptide and the second exon encoding all of the putative neuropeptide products of the precursor protein [18]. It is also evident that in all three echinoderm species the L-type SALMFamide precursor protein is shorter than the F-type SALMFamide precursor protein (Figure 4), which may also therefore be a feature of SALMFamide precursors in other echinoderms. In addition to these general characteristics of SALMFamide precursors, more detailed comparative analysis of the sequence data provides further insights on the structural characteristics and diversity of SALMFamide neuropeptides and the organisation of the precursor proteins that they are derived from, as discussed below.

### Comparative Analysis of the Sequences of L-type SALMFamide Precursors and Putative SALMFamides Derived from L-type SALMFamide Precursors

**Figure 5:**
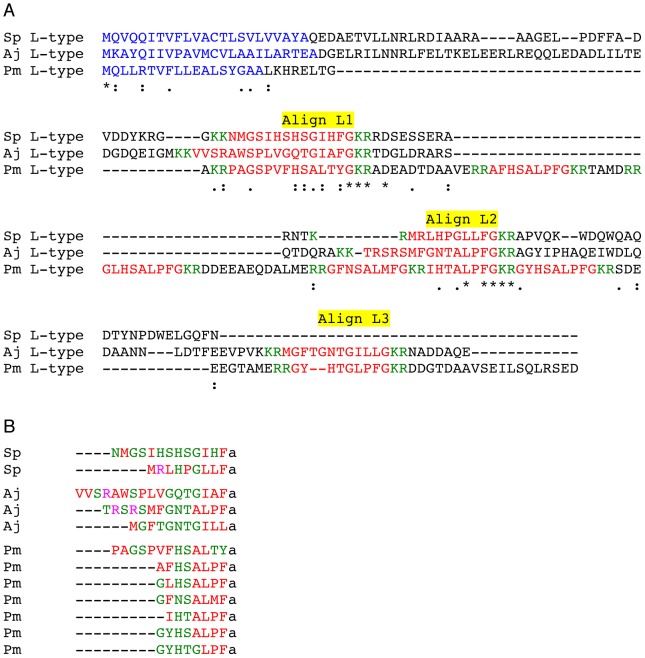
Comparative analysis of L-type SALMFamide precursors and putative SALMFamides derived from L-type SALMFamide precursors. **A.** Multiple sequence alignment of L-type SALMFamide precursors from *Strongylocentrotus purpuratus* (Sp), *Apostichopus japonicus* (Aj) and *Patiria miniata* (Pm). The symbol * labels the positions of residues that are identical in all three sequences, whilst the symbols : and. label the positions of strongly and weakly conserved residues, respectively. Putative neuropeptides that are aligned in all three sequences or that are aligned in the Pm sequence and the Sp or Aj sequences are labelled: Align L1, Align L2 and Align L3. **B.** Comparison of the C-terminally aligned sequences of putative SALMFamides derived from the L-type SALMFamide precursors in Sp, Aj and Pm. Hydrophobic residues are shown in red, hydrophilic residues are shown in green and basic residues are shown in pink. The putative C-terminal amide group is denoted as “a”.

A Clustal Omega alignment of L-type SALMFamide precursors from *S. purpuratus*, *A. japonicus* and *P. miniata* is shown in Figure 5A and regions of the precursor proteins that correspond with putative neuropeptides that are aligned in all three species or that are aligned between *P. miniata* and *S. purpuratus* or *A. japonicus* are labelled Align L1, Align L2 and Align L3. The logic here is that neuropeptides that only align between *S. purpuratus* and *A. japonicus* may be unique to the echinoid/holothurian clade whereas neuropeptides that align in all three species or align between *P. miniata* and *S. purpuratus* or *A. japonicus* could be conserved features of an ancestral L-type SALMFamide precursor.

The putative neuropeptides labelled by Align L1 are between 13 and 17 residues in length (excluding the C-terminal glycine residues that are putative amidation substrates). Furthermore, it is noteworthy that the putative neuropeptides from *A. japonicus* and *P. miniata* in Align L1 both have either one or two proline residues, which due to their conformational rigidity may strongly influence secondary structure characteristics of these peptides [25].

The putative neuropeptides labelled by Align L2 are between 7 and 14 residues in length. Furthermore, it is noteworthy that the putative neuropeptides from *A. japonicus* and *P. miniata* in Align L2 both have the C-terminal sequence TALPFamide.

Align L3 labels putative neuropeptides located in the C-terminal regions of the L-type SALMFamide precursors from *A. japonicus* and *P. miniata*, which are 11 and 8 residues in length, respectively. The *A. japonicus* peptide in Align L3 (MGFTGNTGILLamide) is unusual in having the C-terminal sequence ILLamide but in common with the *P. miniata* peptide in Align L3 (GYHTGLPFamide), it has a Thr-Gly (TG) motif.

Based on the multiple sequence alignment in Figure 5A and the observations above, we speculate that an ancestral L-type SALMFamide precursor may have comprised three L-type SALMFamides corresponding with Align L1, Align L2 and Align L3 in Figure 5A. Thus, the *A. japonicus* L-type SALMFamide precursor conforms most closely with this hypothetical ancestral precursor. Accordingly, we speculate that in echinoids (*S. purpuratus*) the Align L3 peptide was lost and in asteroids multiple intragenic duplication events gave rise to the seven L-type SALMFamides found in the *P. miniata* L-type SALMFamide precursor.

Fig. 5B shows a C-terminal alignment of all of the putative neuropeptides derived from the L-type SALMFamide precursors in *S. purpuratus*, *A. japonicus* and *P. miniata*, with colour-coding according to amino acid properties. What is apparent is that there are clusters of hydrophobic and hydrophilic residues, with 3–4 hydrophobic residues at the C-terminus preceded by a cluster of 2–5 hydrophilic residues. Furthermore, it is noteworthy that basic residues, if present, only occur in the N-terminal regions of putative neuropeptides.

### Comparative Analysis of the Sequences of F-type SALMFamide Precursors and Putative SALMFamides Derived from F-type SALMFamide Precursors

**Figure 6:**
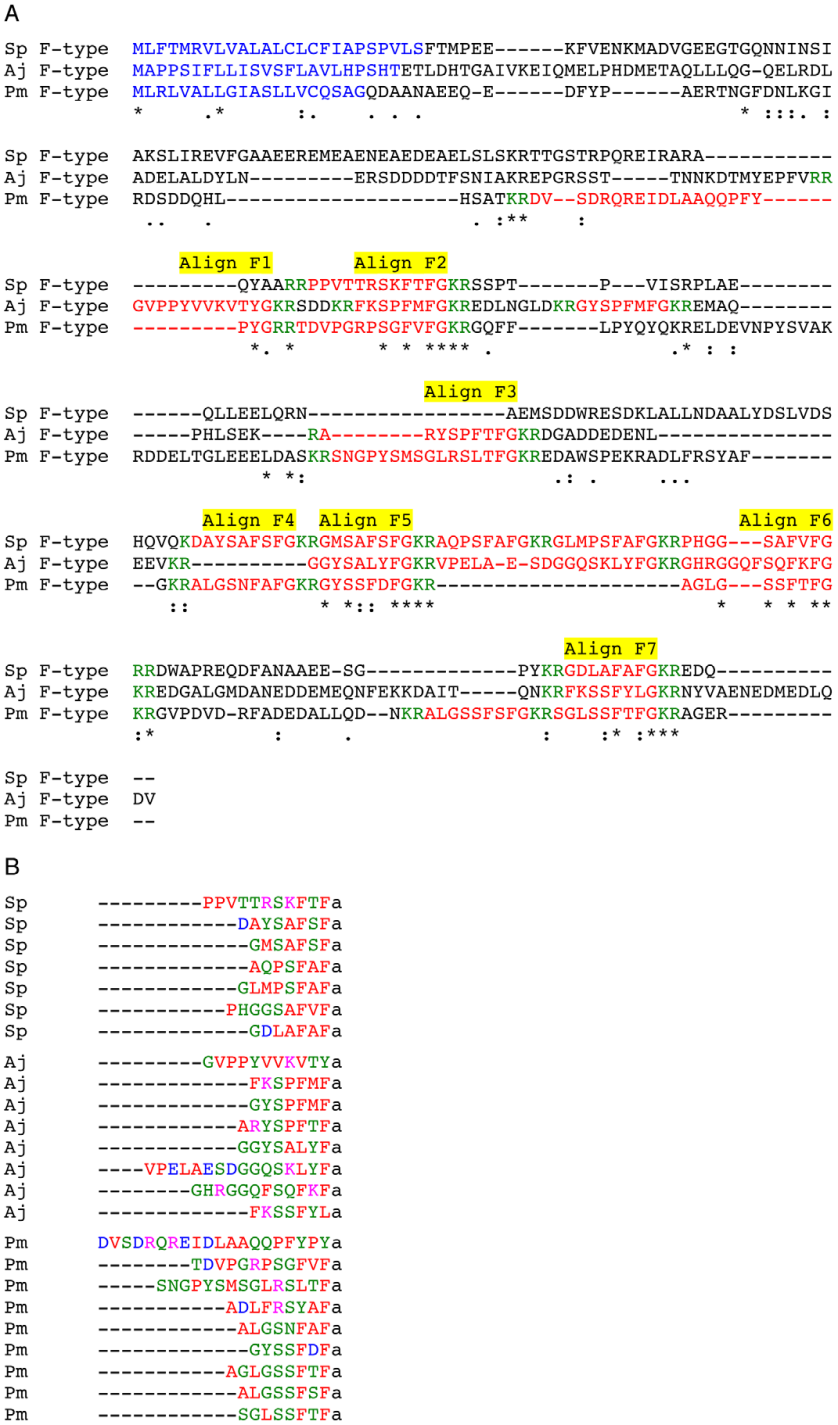
Comparative analysis of F-type SALMFamide precursors and putative SALMFamides derived from F-type SALMFamide precursors. A. Multiple sequence alignment of F-type SALMFamide precursors from *Strongylocentrotus purpuratus* (Sp), *Apostichopus japonicus* (Aj) and *Patiria miniata* (Pm). The symbol * labels the positions of residues that are identical in all three sequences, whilst the symbols : and. label the positions of strongly and weakly conserved residues, respectively. Putative neuropeptides that are aligned in all three sequences or that are aligned in the Pm sequence and the Sp or Aj sequences are labelled: Align F1– AlignF7. **B.** Comparison of the C-terminally aligned sequences of putative SALMFamides derived from the F-type SALMFamide precursors in Sp, Aj and Pm. Hydrophobic residues are shown in red, hydrophilic residues are shown in green, acidic residues are shown in blue and basic residues are shown in pink. The putative C-terminal amide group is denoted as “a”.

A Clustal Omega alignment of F-type SALMFamide precursors from *S. purpuratus*, *A. japonicus* and *P. miniata* is shown in Figure 6A and putative neuropeptides that are aligned in all three species or that are aligned between *P. miniata* and *S. purpuratus* or *A. japonicus* are labelled Align F1– Align F7. As highlighted above for L-type SALMFamide precursors, the logic of this is that neuropeptides that only align between *S. purpuratus* and *A. japonicus* may be unique to the echinoid/holothurian clade whereas neuropeptides that align in all three species or align between *P. miniata* and *S. purpuratus* or *A. japonicus* could be conserved features of an ancestral F-type SALMFamide precursor.

The putative neuropeptides in Align F1 are located in the N-terminal region of the *A. japonicus* and *P. miniata* F-type SALMFamide precursors and are F-type-like SALMFamides with the C-terminal sequences VTYamide and YPYamide, which share a C-terminal Yamide motif. Interestingly, both peptides contain one or two proline residues and in this respect they are similar to the putative neuropeptides in Align L1 of the L-type SALMFamide precursors. Accordingly, the presence of these proline residues may influence secondary structure characteristics of these peptides [25].

The putative neuropeptides in Align F2 are conserved in all three species and have the canonical F-type SALMFamide C-terminal motif FxFamide. Furthermore, two of the neuropeptides have two proline residues: PPVTTRSKFTFamide in *S. purpuratus* and TDVPGRPSGFVFamide in *A. japonicus*. In this respect they are similar to peptides in Align F1 (Figure 6A) and in Align L1 (Figure 5A), with a concordant potential for the proline residues to influence secondary structure characteristics of these peptides [25].

The putative neuropeptides in Align F3 are present in *A. japonicus* (ARYSPFTFamide) and *P. miniata* (SNGPYSMSGLRSLTFamide) but not *S. purpuratus*. Interestingly, the *P. miniata* peptide is an L-type and S2-like SALMFamide, which shares a C-terminal TFamide motif with the *A. japonicus* F-type peptide.

The putative neuropeptides in Align F4 are present in *S. purpuratus* and *P. miniata* but not in *A. japonicus* and they both have the canonical F-type SALMFamide C-terminal motif FxFamide.

The putative neuropeptides in Align F5 are represented in all three species but, interestingly, the *A. japonicus* peptide is an L-type SALMFamide (GGYSALYFamide), whilst the neuropeptides in *S. purpuratus* and *P. miniata* are both canonical F-type SALMFamides with a C-terminal FxFamide motif.

The putative neuropeptides in Align F6 are represented in all three species and are canonical F-type SALMFamides with a C-terminal FxFamide motif.

The putative neuropeptides in Align F7 are represented in all three species and the *S. purpuratus* and *P. miniata* peptides have a canonical F-type SALMFamide FxFamide motif. The *A. japonicus* peptide in Align F7 is unusual in having a C-terminal leucine residue (FKSSFYLamide) and it is noteworthy that it has in common with the *P. miniata* peptide (SGLSSFTFamide) a Ser-Ser motif.

Based on the multiple sequence alignment in Figure 6A and the observations above, we speculate that an ancestral F-type SALMFamide precursor may have comprised seven SALMFamide neuropeptides corresponding with Align F1– Align F7 in Figure 6A. Subsequently, lineage specific intragenic duplication events may have given rise to the additional neuropeptides that fall outside regions labeled by Align F1 to Align F7. An important observation from the data shown in Figure 6A and discussed above is that L-type SALMFamides are not aligned in the *P. miniata* and *A. japonicus* F-type SALMFamide precursors. This suggests that L-type SALMFamides may have arisen independently in the F-type SALMFamide precursors of asteroids (*P. miniata*) and holothurians (*A. japonicus*).

Fig. 6B shows a C-terminal alignment of all of the putative neuropeptides derived from the F-type SALMFamide precursors in *S. purpuratus*, *A. japonicus* and *P. miniata*, with colour-coding according to amino acid properties. As with the peptides derived from L-type SALMFamide precursors (Figure 5B), the 3–4 residues located at the C-terminus of the peptides are predominantly hydrophobic. However, by comparison with peptides derived from L-type SALMFamide precursors, hydrophilic residues are more prevalent at the penultimate amino acid position. In addition, basic and acidic residues are generally more prevalent than in peptides derived from L-type SALMFamide precursors.

### Functional Significance of the Discovery that in the Starfish *P. miniata* S1 is Derived from a Precursor that Comprises Six Other L-type SALMFamides

The L-type SALMFamide precursor identified here in *P. miniata* is noteworthy by comparison with the L-type SALMFamide precursors in *A. japonicus* and *S. purpuratus* because it comprises more putative neuropeptides. Thus, in addition to S1, there are six other putative L-type neuropeptides. Evidence that multiple L-type SALMFamides exist in starfish has, however, been reported previously [20]. Whilst only S1 and S2 were isolated from *A. rubens* and *A. forbesi*, when extracts of radial nerve cords from *M. glacialis* were analysed using HPLC and radioimmunoassays employing antibodies to S1 and S2, other L-type peptides were discovered. In addition to S1, which has thus far been found in all starfish species analysed [7], [20], three other peptides were isolated from *M. glacialis*: an S2-like peptide (SGPYSMTSGLTFamide; MagS2), Mag S3 (AYHSALPFamide) and MagS4 (AYQTGLPFamide) [20]. With the identification of a gene encoding the L-type SALMFamide precursor in *P. miniata* it is evident that the *M. glacialis* neuropeptides MagS3 and MagS4 are similar to two of the L-type SALMFamides in *P. miniata*: AFHSALPFamide and GYHTGLPFamide, respectively. Furthermore, in addition to these two peptides, there are three other putative neuropeptides derived from the *P. miniata* L-type SALMFamide precursor that have the C-terminal motif LPFamide. Lastly, there is also an N-terminally extended putative L-type neuropeptide with two proline residues (PAGSPVFHSALTYamide).

Investigation of the physiological roles of S1 in starfish has revealed that this peptide has inhibitory actions. Thus, it causes relaxation of three preparations from *A. rubens*: the cardiac stomach, tube feet and apical muscle [9], [10]. In addition, experimental studies on the starfish *Asterina pectinifera* have revealed that S1 causes inhibition of potassium-induced release of gonad-stimulating hormone from radial nerves [26]. We now know, however, based on analysis of *P. miniata* genome sequence data, that S1 is co-synthesized and presumably co-released with six other L-type SALMFamides. This therefore raises questions with regard to the actions of these other L-type SALMFamides. Do they, like S1, also have inhibitory actions? Preliminary data obtained from experiments testing the effects of the *M. glacialis* L-type SALMFamide MagS3 on cardiac stomach preparations from *A. rubens* have revealed that, like S1, it causes relaxation of the cardiac stomach. However, when tested at a concentration of 1 µM MagS3 was less effective than S1 as a muscle relaxant in *A. rubens* [20]. Clearly, to gain a deeper insight on the functional significance of the existence of the seven L-type SALMFamides derived from the *P. miniata* L-type precursor, further experimental studies will now need to be conducted, testing and comparing the effects of all seven peptides on preparations from *P. miniata*.

### Functional Significance of the Discovery of an F-type SALMFamide Precursor in *P. miniata*

F-type SALMFamides with the C-terminal motif FxFamide (GYSPFMFamide and FKSPFMFamide) were first isolated from the sea cucumber *A. japonicus* on account of their relaxing actions on muscle preparations [16]. We now know that these two peptides are derived from an F-type SALMFamide precursor that comprises other F-type SALMFamides and three L-type or L-type-like SALMFamides ([19]; Figure 4C). Furthermore, the identification of an F-type SALMFamide precursor in the sea urchin *S. purpuratus* revealed that F-type SALMFamides are not only found in holothurians but also occur in their sister group – the echinoids [17]. With the identification of an F-type SALMFamide precursor in the starfish *P. miniata*, it has been established that the phylogenetic distribution of F-type SALMFamides extends to the asteroids and therefore their evolutionary origin can be traced back at least as far as the common ancestor of echinoids, holothurians and asteroids. However, nothing is known about the pharmacological actions and physiological roles of F-type SALMFamides in starfish or in sea urchins. Based on what is known about the pharmacological effects of F-type SALMFamides as muscle relaxants in sea cucumbers [16], it seems likely that F-type SALMFamides also have inhibitory effects in starfish and sea urchins. However, it remains to be determined what is the physiological relevance of the existence of two types of SALMFamides (L-type and F-type) in echinoderms. Are they expressed by distinct or overlapping populations of neurons? Indirect evidence from immunocytochemical studies [12], [13] suggests that L-type and F-type SALMFamide precursor genes are expressed by different populations of neurons in starfish, as discussed below. However, definitive investigation of this issue will require analysis using double-labelling mRNA *in situ* hybridisation techniques.

Lastly, nothing is known about the receptor(s) that mediate the effects of SALMFamides in echinoderms. Do F-type SALMFamides and L-type SALMFamides exert effects by binding to different receptor types? With the growing body of genomic/transcriptomic sequence data obtained from several echinoderm species it may now be possible to address this issue, probably by working with the assumption that SALMFamide receptors are likely to be G-protein coupled receptors.

### Functional Significance of the Discovery that an S2-like Peptide is Derived from an F-type SALMFamide Precursor in *P. miniata*

S1 (GFNSALMFamide) and S2 (SGPYSFNSGLTFamide) share the C-terminal sequence FNSxLxFamide (where x is variable), and therefore it was proposed that S1 and S2 may have arisen through processes of intragenic or gene duplication followed by sequence divergence [6]. With the development of antibodies to S1 and S2 it became possible to investigate the expression of these two peptides in starfish. Interestingly, whilst the overall tissue/organ distribution of S1 and S2 is very similar, at the cellular/sub-cellular level double-labelling immunocytochemistry revealed that S1 and S2 appear to be expressed by different populations of neurons [12], [13]. Therefore, these data indicated that S1 and S2 are not derived from the same precursor but are derived either from alternatively spliced products of the same gene or from products of two different genes. With the identification of SALMFamide genes in *P. miniata* we now know that S1 and the S2-like peptide SNGPYSMSGLRSLTFamide are encoded by different genes, with S1 being one of seven putative neuropeptide products of the L-type SALMFamide precursor and the S2-like peptide being the one and only L-type peptide that is derived from the F-type SALMFamide precursor in this species. Accordingly, it seems likely that in *Asterias rubens* and *Asterias forbesi* S1 is derived from a L-type SALMFamide precursor and S2 is derived from an F-type SALMFamide precursor. This is interesting from a functional perspective because S1 and S2 share the common C-terminal motif FNSxLxFamide (where x is variable). Therefore, this suggests that peptides (S1 and S2) derived from different SALMFamide precursors may have acquired a common motif by convergent molecular evolution. Furthermore, it is noteworthy that S2 is more potent/effective than S1 as a muscle relaxant in starfish [9], [10], [15]. What might be the functional significance of this? A clue may be found in the observation that S2, but not S1, can be detected by radioimmunoassay in the perivisceral coelomic fluid of the starfish *Asterias rubens* [11]. This suggests that S2 may act as a hormone in starfish, which may also explain why S2 is more potent than S1. In this context, the discovery that an S2-like peptide is derived from the F-type SALMFamide precursor in *P. miniata* is intriguing. Thus, do F-type SALMFamides act as hormones in starfish? Furthermore, is the occurrence of L-type SALMFamides in F-type SALMFamide precursors a feature that enables convergent activation of L-type SALMFamide receptors by peptides derived from different precursors and acting via different signalling pathways: i.e. hormonal (S2 or S2-like) versus non-hormonal (S1). Clearly, these are speculative hypotheses that will require detailed experimental analysis. However, they provide a framework for investigation of the physiological relevance of the occurrence of L-type SALMFamides in F-type SALMFamide precursors.

### The Physiological Significance of Neuropeptide “Cocktails” Derived from the Same Precursor: the SALMFamides as a Model Experimental System

The identification of SALMFamide genes in echinoderms has revealed a remarkable diversity of SALMFamide neuropeptides in each species. Thus in starfish, from the original biochemical identification of two SALMFamides (S1 and S2) in *Asterias* we have progressed to the discovery of sixteen putative SALMFamides in *P. miniata* based on analysis of the sequences of its two SALMFamide genes. But what is the physiological significance of the multiple neuropeptides that are derived from each of the two SALMFamide precursors? This is a question that remains largely unresolved, not only for SALMFamide precursors in echinoderms but for neuropeptide precursors in general. The occurrence of neuropeptide precursors comprising multiple isoforms of a particular neuropeptide type is a common phenomenon, particularly in invertebrates [4], [27], [28]. Furthermore, its physiological relevance has been investigated, most notably in model invertebrates such as *Drosophila melanogaster*. For example, the actions of eight FMRFamide-related neuropeptides that are derived from the same precursor were examined by testing and comparing their effects on nerve-stimulated contraction of larval body-wall muscles [29]. Seven of the peptides strongly enhanced twitch tension and one of the peptides was inactive. The potency and efficacy of the seven active peptides were similar and when the peptides were tested in combination at ratios corresponding to copy number in the precursor protein, their effects were additive and there was no evidence of higher-order interactions among them. It was concluded, therefore, that with respect to effects on nerve-stimulated contraction of *Drosophila* larval body-wall muscles the seven active peptides exhibit functional redundancy. However, the authors of this study cautiously highlight the need to “search for additional functions or processes in which these peptides may act differentially” [29].

Evidence that structurally-related neuropeptides derived from the same precursors are not simply functionally redundant isoforms has been provided by detailed analysis of genome sequence data comparing the neuropeptidome of twelve species belonging to the genus *Drosophila*, the common ancestor of which is estimated to date back 40–60 million years. Remarkably, the neuropeptidome was found to be identical in all twelve *Drosophila* species [30]. This finding indicates that the multiple isoforms of neuropeptides derived from the same precursor are under stabilizing selection and therefore are unlikely to be functionally redundant. These important findings from comparative genomic analyses highlight the need now to investigate and compare in greater detail the pharmacological actions and physiological roles of neuropeptide isoforms derived from the same precursor protein.

Our discovery of SALMFamide genes in echinoderms has highlighted new model systems in which the physiological relevance of structurally related neuropeptide isoforms derived from the same precursor could be investigated. Of particular interest in this regard are the F-type SALMFamide precursors in the sea cucumber *A. japonicus* and the starfish *P. miniata*, which largely comprise F-type or F-type-like SALMFamides but which also contain one or more L-type SALMFamides. The functional significance of these neuropeptide “cocktails” could be investigated using established *in vitro* and *in vivo* assays [9], [10].

## Concluding Comments

The data presented in this paper have provided new insights on the evolution and diversity of SALMFamide neuropeptides in the Phylum Echinodermata and further insights will be obtained when genome and/or neural transcriptome data are available for ophiuroid and crinoid species. However, currently nothing is known about relationships between SALMFamide neuropeptides in echinoderms and neuropeptides in other phyla. Do SALMFamide neuropeptides also exist in hemichordates, a sister phylum to the echinoderms? Genome sequence data are available for the hemichordate species *Saccoglossus kowalevskii* (https://www.hgsc.bcm.edu/content/acorn-worm-genome-project) but thus far our analysis of these data has not revealed genes encoding peptides that resemble SALMFamide neuropeptides. If the receptors that mediate the effects of SALMFamide neuropeptides in echinoderms can be identified, then this may reveal relationships between SALMFamides and neuropeptides in other phyla.
